# Reproducibility of domain-specific physical activity over two seasons in children

**DOI:** 10.1186/s12889-018-5743-8

**Published:** 2018-07-03

**Authors:** Eivind Aadland, Lars Bo Andersen, Ulf Ekelund, Sigmund Alfred Anderssen, Geir Kåre Resaland

**Affiliations:** 1 Department of Sport, Food and Natural Sciences, Faculty of Education, Arts and Sports, Western Norway University of Applied Sciences, Campus Sogndal, Box 133, 6851 Sogndal, Norway; 2 Department of Sports Medicine, Norwegian School of Sport Sciences, Oslo, Norway; 3 Center for Health Research, Førde Central Hospital, Førde, Norway

**Keywords:** Test-retest, Reliability, Agreement, Intra-class correlation, Measurement, Accelerometry

## Abstract

**Background:**

Knowledge of the reproducibility of domain-specific accelerometer-determined physical activity (PA) estimates are a prerequisite to conduct high-quality epidemiological studies. The aim of this study was to determine the reproducibility of objectively measured PA level in children during school hours, afternoon hours, weekdays, weekend days, and total leisure time over two different seasons.

**Methods:**

Six hundred seventy six children from the Active Smarter Kids study conducted in Sogn og Fjordane, Norway, were monitored for 7 days by accelerometry (ActiGraph GT3X+) during January–February and April–May 2015. Reproducibility was estimated week-by-week using intra-class correlation (ICC) and Bland-Altman plots with 95% limits of agreement (LoA).

**Results:**

When controlling for season, reliability (ICC) was 0.51–0.66 for a 7-day week, 0.55–0.64 for weekdays, 0.11–0.43 for weekend days, 0.57–0.63 for school hours, 0.42–0.53 for afternoon hours, and 0.42–0.61 for total leisure time. LoA across models approximated a factor of 1.3–2.5 standard deviations of the sample PA levels. 3–6 weeks of monitoring were required to achieve a reliability of 0.80 across all domains but weekend days, which required 5–32 weeks.

**Conclusion:**

Reproducibility of PA during leisure time and weekend days were lower than for school hours and weekdays, and estimates were lower when analyzed using a week-by-week approach over different seasons compared to previous studies relying on a single short monitoring period. To avoid type 2-errors, researchers should consider increasing the monitoring period beyond a single 7-day period in future studies.

**Trial Registration:**

ClinicalTrials.gov, NCT021324947. Registered on 7 April 2014.

**Electronic supplementary material:**

The online version of this article (10.1186/s12889-018-5743-8) contains supplementary material, which is available to authorized users.

## Background

There are many unresolved issues regarding data reduction and quality assessment of accelerometry data to determine physical activity (PA) and sedentary behavior (SED). These challenges leads to great variation in procedures used and criteria applied to define a valid measurement [1]. As behavior vary greatly over time, an important aspect of accelerometer measurements is how many days or periods of measurement that need to be included to obtain reliable estimates of habitual activity level. This is particularly true when children live in an area with a significant change in weather during different seasons [2–4]. Measurement error caused by poor reproducibility (amongst other sources of error) may preclude researchers from arriving at valid conclusions and possibly misinform the society regarding PA as a target for public health initiatives [5].

Most studies in children apply a criterion of a minimum 3 or 4 wear days to constitute a reproducible accelerometer-measurement [1]. Although findings vary between studies in both adults [6–10] and children [11–22], most evidence suggest that a reasonable reliability (i.e., intra-class correlation (ICC)) of ~ 0.70–0.80 are achieved with 3–7 days of monitoring. However, the reproducibility might vary across PA domains, as suggested by a study in adults [7]. Given the importance of evaluating effects of interventions in preschool [23, 24] and school settings [25, 26], especially considering that there might be a reactivity effect for PA [27], it is critical to determine activity level during school hours and leisure time separately. Moreover, many studies target in-school versus out-of-school, or weekday versus weekend activity patterns [28–31]. The validity of such studies depends on whether the intended associations, patterns or effects can be reliably captured during the applied monitoring period.

Most previous studies have estimated the reliability and the number of days needed based on the Spearman Brown prophecy formula for measurements conducted over a single 7-day period [8, 9, 11, 13–22]. Such study designs have received critique for being likely to underestimate the number of days needed, and they should therefore be interpreted with caution [32–34]. Importantly, such results are in principle only generalizable to the included days, as inclusion of additional days, weeks or seasons will add variability. Few studies have determined the reliability for longer periods, of which all have shown considerable intra-individual variation [34–38]. Reliability has been shown to be ~ 0.70–0.80 with limits of agreement (LoA) ~ 1 standard deviation (SD) for one out of two and three consecutive weeks of measurement in preschool children and adults, respectively [37, 38]. However, poorer estimates are found in studies considering seasonality [34–36], leaving reliability estimates of ~ 0.50 for one week monitoring in children [34, 36]. These findings agrees with studies showing substantial seasonal variation in activity level in children and adolescents [2–4], which are obviously not captured when relying on a single measurement period. Finally, agreement (i.e., LoA and/or standard error of the measurement), which provide researchers a direct quantification of how much outcomes should be expected to vary over time [39–41], has not been reported for domain-specific PA or SED in children.

The aim of the present study was to determine the domain-specific reproducibility of accelerometer-determined PA and SED for one week of measurement obtained during two different seasons separated by 3–4 months in a large sample of children. We hypothesized great variability across weeks and reliability estimates lower than ICC = 0.80 for all accelerometer outcomes, but somewhat better reliability for school hours compared to leisure time.

## Methods

### Participants

The present analyses are based on data obtained in fifth grade children from the Active Smarter Kids (ASK) cluster-randomized trial, conducted in Norway during 2014–2015 [26, 42]. The main aim of the ASK study was to investigate the effect of school-based PA on academic performance and various health outcomes. Physical activity was measured with accelerometry at baseline (mainly May to June 2014) and follow-up (April to May 2015) in all children, as well as in approximately two-thirds of the children that we invited to complete a mid-term (January to February 2015) measurement. In the present study, we include the mid-term and the follow-up measurement, to allow for comparison of PA over two different seasons separated by 3–4 months. Additionally, as the intervention was ongoing at both these time-points, and we found no effect of the intervention on PA [26], we included both the intervention and the control groups. We have previously published a detailed description of the study [42], and do only provide a brief overview of the accelerometer handling herein.

Our procedures and methods conform to ethical guidelines defined by the World Medical Association’s Declaration of Helsinki and its subsequent revisions. The Regional Committee for Medical Research Ethics approved the study protocol. We obtained written informed consent from each child’s parents or legal guardian and from the responsible school authorities prior to all testing. The study is registered in Clinicaltrials.gov with identification number: NCT02132494.

### Procedures

Physical activity was measured using the ActiGraph GT3X+ accelerometer (Pensacola, FL, USA) [43]. During both measurements, participants were instructed to wear the accelerometer at all times over 7 consecutive days, except during water activities (swimming, showering) or while sleeping. Units were initialized at a sampling rate of 30 Hz. Files were analyzed at 10 s epochs using the KineSoft analytical software version 3.3.80 (KineSoft, Loughborough, UK). In all analyses, consecutive periods of ≥20 min of zero counts were defined as non-wear time [1, 44]. Results are reported for overall PA level (cpm), as well as minutes per day spent SED (< 100 cpm), in light PA (LPA) (100–2295 cpm), in moderate PA (MPA) (2296–4011 cpm), in vigorous PA (VPA) (≥ 4012 cpm), and in moderate-to-vigorous PA (MVPA) (≥ 2296 cpm), determined using previously established and validated cut points [45, 46].

Results were reported for a 7-day week (mean of up to 7 days providing ≥8 h of wear time), weekdays (mean of up to 5 days providing ≥8 h of wear time), weekend days (mean of up to 2 days providing ≥8 h of wear time), school hours (mean of up to 5 days providing ≥4 h of wear time), afternoon (mean of up to 5 days providing ≥4 h of wear time), and total leisure time (mean of afternoon and weekend days). Data for a full day was restricted to hours 06:00 to 23:59, whereas we defined school hours from 09:00 to 14:00, and afternoon from 15:00 to 23.59. We only included children that provided data for all domains for analysis, that is, ≥ 3 weekdays, school days and afternoons, as well as ≥1 weekend day. We also conducted sensitivity analyses restricted to children providing ≥4 weekdays, school days and afternoons, and 2 weekend days.

### Statistical analyses

Children’s characteristics were reported as frequencies, means and SDs. Differences between included and excluded children and differences in PA and SED between measurements was tested using a mixed effect model including random intercepts for children. Wear time was included as a covariate for analyses of PA and SED. Differences in PA and SED between measurements were reported as standardized effect sizes (ES = mean difference between seasons/square root((SD_winter_^2^ + SD_spring_^2^/2))).

We estimated reproducibility using a week-by-week approach [37, 38]. Reliability for a single week of measurement (ICC_s_) was assessed using variance partitioning applying a one-way random effect model not controlling for season (i.e., determining reliability based on an absolute agreement definition) and a two-way mixed effect model controlling for season (i.e., determining reliability based on a consistency definition) [47]. In the latter model, the variance attributed to season is removed, thus, it determines to what degree children retain their rank between the two time points, without respect to the mean difference between the two measurements. All models were adjusted for wear time. Number of weeks needed to obtain a reliability of 0.80 (N) was estimated using the Spearman Brown prophecy formula (ICC for average measurements [ICC_k_]) [6, 47]: N = ICC_t_/(1-ICC_t_)*[(1-ICC_s_)/ICC_s_], where N = the number of weeks needed, ICC_t_ = the desired level of reliability, and ICC_s_ = the reliability for a single week.

We additionally applied Bland Altman plots to assess agreement, showing the difference between two subsequent weeks as a function of the mean of the two weeks [39]. We calculated 95% LoA from the residual variance (i.e., within-subjects) error term based on the variance partitioning models (LoA = √residual variance *√2*1.96) [41].

All analyses were performed using IBM SPSS v. 23 (IBM SPSS Statistics for Windows, Armonk, NY: IBM Corp., USA). A *p*-value < .05 indicated statistically significant findings.

## Results

### Participants’ characteristics

Of the 1129 included children in the ASK-study, 676 provided accelerometer data at the mid-term and post measurement, of whom 465 children aged 10.4 to 11.6 years (69% of the total sample; 48% boys) provided valid data for analyses of PA (Table 1). There were no differences between the included (*n* = 465) and excluded (664) children in anthropometry (*p* ≥ .064). During school hours, the children included in the present analyses were more SED (mean (95% CI) 3.7 (1.5–5.9) min/day, *p* = .001), and demonstrated lower overall (− 41 (− 64–-18) cpm, p = .001) and intensity-specific PA levels (MPA: − 1.7 (− 2.4–-0.9) min/day; VPA: − 1.3 (− 2.1–-0.6) min/day; MVPA: − 3.0 (− 4.3–-1.7) min/day; all *p* ≤ .001), compared to the excluded children. Differences were smaller among other domains.

**Table 1:** The children’s characteristics (*n* = 447–465). Values are mean (SD) if not otherwise stated

	Boys (*n* = 225)	Girls (*n* = 240)	Total (*n* = 465)
Age (years)	10.9 (0.3)	10.9 (0.3)	10.9 (0.3)
Body mass (kg)	39.1 (8.4)	39.3 (8.7)	39.2 (8.5)
Height (cm)	146 (7)	146 (7)	146 (7)
BMI (kg/m^2^)	18.2 (2.9)	18.2 (3.1)	18.2 (3.0)
Overweight/obese (%)	17/4	15/4	16/4

*BMI* Body mass index

In general, overall PA level (cpm) and intensity-specific PA was significantly higher, and SED was significantly lower, in the spring than in the winter (ES = 0.08–0.75 for the total 7-d week) (Table 2; Additional file 4: Table S1). Differences were modest during school hours (ES = 0.01–0.21), but much greater during the afternoon (ES = 0.27–0.87) and during total leisure time (ES = 0.23–0.91). Differences for weekdays (ES = 0.06–0.67) and weekend days (ES = 0.10–0.60) were similar.

**Table 2:** Physical activity level during the winter and spring measurements. Values are mean (SD)

Physical activity level*	7-d Week	Weekdays	Weekend	School	Afternoon	Leisure
	Winter					
Wear days (days/week)	6.5 (0.8)	4.7 (0.6)	1.8 (0.4)	4.5 (0.7)	4.6 (0.6)	6.4 (0.8)
Wear minutes (min/day)	778 (46)	807 (49)	700 (83)	295 (6)	382 (39)	470 (44)
Overall PA (cpm)	481 (125)	510 (134)	406 (163)	582 (167)	438 (167)	429 (147)
SED (min/day)	508 (50)	521 (54)	472 (77)	183 (18)	259 (40)	318 (43)
LPA (min/day)	215 (33)	225 (34)	190 (47)	85 (13)	99 (21)	124 (25)
MPA (min/day)	35 (10)	38 (12)	25 (13)	16 (5)	14 (6)	17 (7)
VPA (min/day)	18 (9)	21 (11)	11 (9)	13 (6)	8 (6)	9 (6)
MVPA (min/day)	53 (18)	59 (20)	36 (21)	26 (10)	22 (11)	26 (12)
	Spring					
Wear days (days/week)	6.4 (0.6)	4.6 (0.6)	1.8 (0.4)	4.4 (0.7)	4.6 (0.6)	6.4 (0.8)
Wear minutes (min/day)	789 (48)	812 (48)	732 (90)	295 (6)	388 (36)	485 (43)
Overall PA (cpm)	617 (204)	630 (200)	580 (368)	621 (201)	667 (295)	643 (268)
SED (min/day)	499 (54)	509 (56)	475 (87)	183 (18)	247 (39)	311 (46)
LPA (min/day)	221 (33)	228 (33)	203 (50)	83 (13)	107 (21)	134 (25)
MPA (min/day)	39 (12)	42 (13)	31 (16)	17 (6)	18 (7)	22 (8)
VPA (min/day)	26 (14)	29 (15)	20 (19)	13 (7)	15 (9)	16 (10)
MVPA (min/day)	66 (24)	71 (25)	51 (32)	28 (11)	33 (15)	38 (17)

*PA* Physical activity; *cpm* Counts per minute; *SED* Sedentary time; *LPA* Light physical activity; *MPA* Moderate physical activity; *VPA* Vigorous physical activity; *MVPA* Moderate-to-vigorous physical activity; all results are based on n = 465 children

### Reproducibility

As PA and SED differed substantially between the two measurements for most outcomes, reliability estimates improved when controlling for season (i.e., applying a consistency definition of reliability) versus when not controlling for season (i.e., applying an absolute agreement definition of reliability). The difference between estimates was greatest for variables having the greatest bias. A 7-d week provided ICC values 0.31–0.66 when not controlling for season, and 0.51–0.66 when controlling for season (Table 3). Reliability estimates for weekdays was very similar to an overall 7-d week, whereas weekend days clearly provided poor reliability estimates whether controlling for season or not (ICC = 0.01–0.43). On the contrary, reliability estimates for school hours was modest, being more or less identical whether controlling for season or not (ICC = 0.56–0.63) (Table 4), due to the small bias. Estimates for afternoon was lower than for school hours, with clear improvements when controlling for season, due to the much larger bias between seasons than for school hours (ICC = 0.15–0.50 when not controlling for season vs. 0.42–0.53 when controlling for season). The total leisure time estimates was similar to the estimates for the afternoon (ICC = 0.14–0.59 and 0.42–0.61 when not controlling and controlling for season, respectively).

**Table 3:** The reproducibility of different outcome variables for one out of two weeks of measurement for a 7-d week, weekdays and weekend days, applying a ≥ 3 weekdays and ≥ 1 weekend day wear time criterion (*n* = 465 (69%) children)

	7-day Week			Weekdays			Weekend days		
	ICC_s_	LoA	N	ICC_s_	LoA	N	ICC_s_	LoA	N
	Not corrected for season (absolute agreement definition)								
Overall PA (cpm)	0.31	421	9.1	0.38	395	6.6	0.01	818	291
SED (min/day)	0.60	74.2	2.6	0.59	78.5	2.8	0.32	134	8.4
LPA (min/day)	0.66	49.5	2.1	0.62	53.3	2.5	0.43	89.2	5.4
MPA (min/day)	0.55	21.2	3.3	0.54	23.1	3.4	0.21	36.0	14.8
VPA (min/day)	0.43	25.9	5.2	0.47	27.4	4.5	0.09	41.6	38.3
MVPA (min/day)	0.52	42.1	3.7	0.54	44.9	3.5	0.16	70.1	21.1
	Corrected for season (consistency definition)								
Overall PA (cpm)	0.51	329	3.9	0.55	316	3.2	0.11	742	31.4
SED (min/day)	0.65	68.3	2.1	0.63	73.5	2.4	0.35	129	7.3
LPA (min/day)	0.66	49.2	2.1	0.62	53.2	2.5	0.43	88.8	5.3
MPA (min/day)	0.59	19.8	2.8	0.58	21.8	2.9	0.24	35.0	12.5
VPA (min/day)	0.61	20.4	2.6	0.62	22.2	2.5	0.19	37.9	17.5
MVPA (min/day)	0.64	34.8	2.2	0.64	38.1	2.2	0.23	65.2	13.5

*PA* Physical activity; *cpm* Counts per minute; *SED* Sedentary time; *LPA* Light physical activity; *MPA* Moderate physical activity; *VPA* Vigorous physical activity; *MVPA* Moderate-to-vigorous physical activity; *ICC*_*s*_ Intra-class correlation for a single week of measurement adjusted for wear time; *N* Number of weeks needed to achieve a ICC = 0.80; *LoA* 95% Limits of agreement. Estimates not corrected for season are derived from one-way random effect models (i.e., using an absolute agreement definition of reproducibility), whereas models corrected for season are derived from two-way mixed effect models (i.e., using a consistency definition of reproducibility)

**Table 4:** The reproducibility of different outcome variables for one out of two weeks of measurement for school hours, afternoon and total leisure time, applying a ≥ 3 weekdays and ≥ 1 weekend day wear time criterion (*n* = 465 (69%) children)

	School hours			Afternoon hours			Leisure time*		
	ICC_s_	LoA	N	ICC_s_	LoA	N	ICC_s_	LoA	N
	Not corrected for season (absolute agreement definition)								
Overall PA (cpm)	0.56	343	3.2	0.15	679	22.8	0.14	621	24.3
SED (min/day)	0.63	30.0	2.4	0.38	63.3	6.5	0.47	66.1	4.6
LPA (min/day)	0.57	22.9	3.0	0.50	38.8	4.0	0.59	41.1	2.8
MPA (min/day)	0.57	10.0	3.0	0.30	16.6	9.3	0.35	17.7	7.3
VPA (min/day)	0.57	12.1	3.0	0.26	19.9	11.6	0.24	21.7	12.5
MVPA (min/day)	0.60	18.3	2.7	0.29	33.5	9.9	0.31	36.0	9.0
	Corrected for season (consistency definition)								
Overall PA (cpm)	0.57	335	3.0	0.42	507	5.6	0.42	462	5.5
SED (min/day)	0.63	30.1	2.4	0.51	54.1	3.9	0.57	57.1	3.0
LPA (min/day)	0.58	22.6	2.9	0.53	37.0	3.5	0.61	39.6	2.5
MPA (min/day)	0.57	10.0	3.0	0.43	14.3	5.2	0.46	15.5	4.7
VPA (min/day)	0.58	12.0	2.9	0.47	15.5	4.5	0.46	16.8	4.6
MVPA (min/day)	0.61	17.8	2.5	0.50	26.1	4.0	0.51	28.3	3.9

*PA* Physical activity; *cpm* Counts per minute; *SED* Sedentary time; *LPA* Light physical activity; *MPA* Moderate physical activity; *VPA* Vigorous physical activity; *MVPA* Moderate-to-vigorous physical activity; *ICC*_*s*_ Intra-class correlation for a single week of measurement adjusted for wear time; *N* Number of weeks needed to achieve a ICC = 0.80; LoA = 95% limits of agreement; *Afternoon + Weekend. Estimates not corrected for season are derived from one-way random effect models (i.e., using an absolute agreement definition of reproducibility), whereas models corrected for season are derived from two-way mixed effect models (i.e., using a consistency definition of reproducibility)

When controlling for season, the number of weeks needed to obtain a reliability of 0.80 as estimated by the Spearman Brown prophecy formula was 2.2–3.9 for an overall 7-d week, 2.2–3.2 for weekdays, 5.3–31.4 for weekend days, 2.4–3.0 for school hours, 3.5–5.6 for afternoon, and 2.5–5.5 for total leisure time. A substantial intra-individual variation over time for all outcomes is also indicated by the LoA (Table 3; Table 4; Additional file 1: Figure S1, Additional file 2: Figure S2 and Additional file 3: Figure S3), which approximated factors of 1.3–1.8, 1.3–1.8, 1.6–2.5, 1.7–1.8, 1.4–2.0, 1.3–1.9 SDs of the sample PA levels, respectively. Overall PA level (cpm) was the least reproducible outcome across models. In general, we found minor improvements in reproducibility when the analyses were restricted to those children (*n* = 257, 38%) providing data for ≥4 weekdays and 2 weekend days (Additional file 5: Table S2).

## Discussion

The aim of the present study was to determine domain-specific reproducibility of accelerometer-determined PA and SED in children over two separate weeks of monitoring undertaken 3–4 months apart. Our results suggest that one week of accelerometer monitoring have poorer reliability than suggested by most previous studies that have relied on a single monitoring period. Further, our findings revealed that reliability was superior during school hours and weekdays as compared to leisure time.

Previous studies conclude that 3–7 monitoring days provide reliable estimates of PA and SED in children [11–22]. These studies have estimated reliability based on day-by-day analyses, typically using a single 7-day monitoring period. In contrast, but consistent with studies including measurements over several seasons [34–36], our findings shows that longer and/or a higher number of monitoring periods are required to estimate PA and SED with acceptable confidence. Mattocks [36] determined overall PA, MVPA and SED over four 7-day periods over approximately one year using the Actigraph 7164 accelerometer in 11–12-year-old children. The ICC for one single period of measurement varied from 0.45 to 0.59 across outcome variables. Wickel & Welk [34] found an ICC of 0.46 for one out of three 7-day periods to assess steps for the Digiwalker pedometer in 80 children aged ~ 10 years. The present findings along with previous findings may question the validity of one week of measurement to determine children’s habitual activity level.

While levels of PA and SED during weekdays and school hours can be estimated with a reliability of 0.80 across 2–3 week-long monitoring periods, 3–6 weeks of measurement are required to achieve this level of precision for afternoon hours and for total leisure time. This finding is consistent with our hypothesis and a previous study in adults [7]. As variation in behavior are restricted by the school curriculum during school hours, the higher reliability compared to leisure time is expected. During school hours, students follow the same schedule, including a roughly similar time spent in for example physical education and recess, during both monitoring periods. Thus, although some variation in levels and types of activities could be expected also during the school day, this variation would be restricted by the applied teaching methods and established curriculum. On the contrary, our findings indicate that children vary their activity levels greatly across season during leisure time, likely according to climate, weather, and daylight. The present study were collected in the Sogn og Fjordane County in western Norway, and we believe several factors might have caused the great variation in leisure time activity level. In the winter (January–February), daylight fade after hours 15:00–17:00, whereas the spring-measurement (April–May) was conducted when there are daylight until bedtime, giving the children a greatly different opportunity to be active outside. Moreover, the weather can be very different, inviting children to play outside in the spring, whereas they are prone to spend their time inside in the winter. There are also large geographic differences between areas. The coast have a mild and wet climate throughout the year, whereas the inland and mountain areas can have a cold winter and generally less precipitation. Thus, children are likely to spend their time in different modes of activity in different locations across the monitoring periods. Because many movement patterns (e.g., swimming, cycling, and skiing) might be poorly captured by accelerometers [48], such variation in preferred activities over time have the potential to greatly influence stability of accelerometer-derived PA and SED levels during leisure time. According to our findings, these (amongst other) sources of variability influence the weekend days the most, for which we found that 5–32 weeks of measurement was required to achieve an ICC = 0.80. Thus, our results suggest that PA and SED on weekend days, whether controlled for season or not, cannot be measured reliably using a feasible protocol. Yet, it should be kept in mind that the comparison of weekend days only include 1 or 2 days, compared to afternoons including 3, 4, or 5 days, thus, a different precision would be expected, despite both domains being leisure time. Finally, the difference in variance between the two monitoring periods (Table 2) could be an explanation for poor to modest reliability estimates, as the statistical model assumes compound symmetry and the ICC are sensitive to asymmetry [47].

As noise in exposure (x) variables will lead to attenuation of regression coefficients (regression dilution bias), and noise in outcome (y) variables will increase standard errors [5], unreliable measures weaken researchers ability to make valid conclusions. In epidemiology, researchers are in general interested in the long-term habitual PA level, not the very recent days. For example, when evaluating school-interventions, we assume a five-day monitoring period provide a reasonable estimate of true PA or SED. In the school context, our results provide support for modest errors, despite estimates being poorer than suggested in many previous studies. On the contrary, studies evaluating home-based interventions or studies investigating patterns of weekday versus weekend, or in-school and out-of-school activity levels are prone to type 2-errors, if relying on a 7-day monitoring period that provide an insufficient snapshot of children’s habitual activity level. Consequently, such studies must be sufficiently powered. Sample size calculations for designs with repeated measurements normally correct expected SDs for the expected correlations between the baseline and follow-up measurements (i.e., studies use SD for change rather than the cross-sectional SD), thus, the sample size needed is less than for performing cross-sectional analyses if the pre-post correlation is > 0.50. Yet, this benefit is not achievable for PA outcomes during leisure time given the present results. These findings are therefore important for informing study designs and sample size calculations, as well as for interpretation of study findings.

Although an increased accelerometer monitoring length improve reproducibility and thus improve validity of study conclusions, given that accelerometry is a valid measure of the outcome, the burden for study participants should be kept minimal to maximize response rate and compliance. Yet, we have previously performed 2- and 3-week monitoring protocols in preschool children and adults, respectively, without any problems regarding compliance [37, 38]. More recently, we have also successfully performed a 2-week monitoring protocol in larger samples of children, adults and older people, demonstrating this protocol’s acceptance in various context. Yet, performing measurements over separate as opposed to consecutive periods might pose an increased burden for participants, as well as for researchers. Importantly, the required monitoring volume is a matter of the research question posed, as population-estimates on a group level requires less precision than individual-level estimates used for correlational analyses [33].

### Strengths and limitations

The main strength of the present study is the inclusion of a large and representative sample of children. As reliability estimates depends on the sample variation [39–41], the reliability estimates presented herein should be generalizable to other large-scale population-based studies. Another strength is inclusion of measurements conducted 3–4 months apart, during two different seasons, as has only been analyzed in a few previous studies in children [34, 36]. This approach extends findings from previous studies in children that have mainly estimated reproducibility over a single short monitoring period [11, 13–22]. A limitation, though, is the inclusion of only two weeks and two seasons, as inclusion of more observations probably would introduce more variability and lead to more conservative reproducibility estimates [34, 36]. Moreover, Norway has profound seasonal differences in weather conditions. This characteristic might limit generalizability to areas with less pronounced seasonality. Finally, the inclusion of the intervention group in the current analyses might have caused additional variation to the data, as the intervention group participants could be expected to change their PA level over time. Yet, the intervention was ongoing during both measurements, there was no effect of the intervention on PA levels [26], and reproducibility estimates differed marginally and non-systematically between the intervention and control groups. The maximum ICC difference between groups across the variables analyzed were 0.09 for a 7-day week, 0.06 for weekdays, 0.14 for weekend days, 0.06 for school hours, 0.07 for afternoon hours, and 0.09 for total leisure time (results not shown).

## Conclusion

We conclude that the reproducibility for one week of accelerometer monitoring is poor to modest across different domains of PA when seasonal differences is considered. Reliability for a 7-day period was lower than in most previous studies relying on a single monitoring period, and reliability for leisure time and weekend days was lower than for school hours and week days. Longer or repeated measurement periods are favourable compared with one single 7-day period when assessing PA and SED by accelerometry, as this will reduce the possibility of type 2-errors in future studies.

## Additional files

Additional file 1: **Figure S1.** Bland Altman plots of agreement for overall physical activity level (cpm) for different domains over two consecutive weeks of measurement. Bland Altman plots (mean of two weeks of measurement on the x-axis versus the difference between them on the y-axis) for a 7-day week, weekdays, weekend days, school hours, afternoon and total leisure time. All results are based on *n* = 465 children. The full line is the bias between weeks, whereas the dotted lines are 95% limits of agreement. (TIFF 4673 kb)

Additional file 2: **Figure S2.** Bland Altman plots of agreement for sedentary time (min/day) for different domains over two consecutive weeks of measurement. Bland Altman plots (mean of two weeks of measurement on the x-axis versus the difference between them on the y-axis) for a 7-day week, weekdays, weekend days, school hours, afternoon and total leisure time. All results are based on *n* = 465 children. The full line is the bias between weeks, whereas the dotted lines are 95% limits of agreement. Please be aware that variability is not directly comparable between full days and part of days, due to different wear time. (TIFF 4520 kb)

Additional file 3: **Figure S3.** Bland Altman plots of agreement for moderate-to-vigorous physical activity (min/day) for different domains over two consecutive weeks of measurement. Bland Altman plots (mean of two weeks of measurement on the x-axis versus the difference between them on the y-axis) for a 7-day week, weekdays, weekend days, school hours, afternoon and total leisure time. All results are based on n = 465 children. The full line is the bias between weeks, whereas the dotted lines are 95% limits of agreement. Please be aware that variability is not directly comparable between full days and part of days, due to different wear time. (TIFF 5467 kb)

Additional file 4: **Table S1.** Differences (spring – winter) in physical activity level between seasons across all physical activity outcomes. (DOCX 16 kb)

Additional file 5: **Table S2a.** The reliability for different outcome variables for one out of two weeks of measurement for a 7-d week, weekdays and weekend days, applying a ≥ 4 weekdays and 2 weekend days wear time criterion (*n* = 257 (38%) children). **Table S2b.** The reliability for different outcome variables for one out of two weeks of measurement for school hours, afternoon and total leisure time, applying a ≥ 4 weekdays and 2 weekend days wear time criterion (n = 257 (38%) children). (DOCX 20 kb)

## Abbreviations

CI: Confidence interval
CPM: Counts per minute
ICC: Intra-class correlation
ICC_k_: Intra-class correlation for average measurements
ICC_s_: Intra-class correlation for a single week of measurement
ICC_t_: Desired intra-class correlation
LoA: Limits of agreement
LPA: Light physical activity
MPA: Moderate physical activity
MVPA: Moderate-to-vigorous physical activity
n: Number of participants
N: Number of weeks needed to achieve a ICC = 0.80
PA: Physical activity
SD: Standard deviation
SED: Sedentary time
VPA: Vigorous physical activity
