# Supplementary figures and images for: Reproducibility of domain-specific physical activity over two seasons in children
Source: BMC Public Health. 2018 Jul 3;18:821. doi: 10.1186/s12889-018-5743-8 (PMC6029381; doi:10.1186/s12889-018-5743-8)

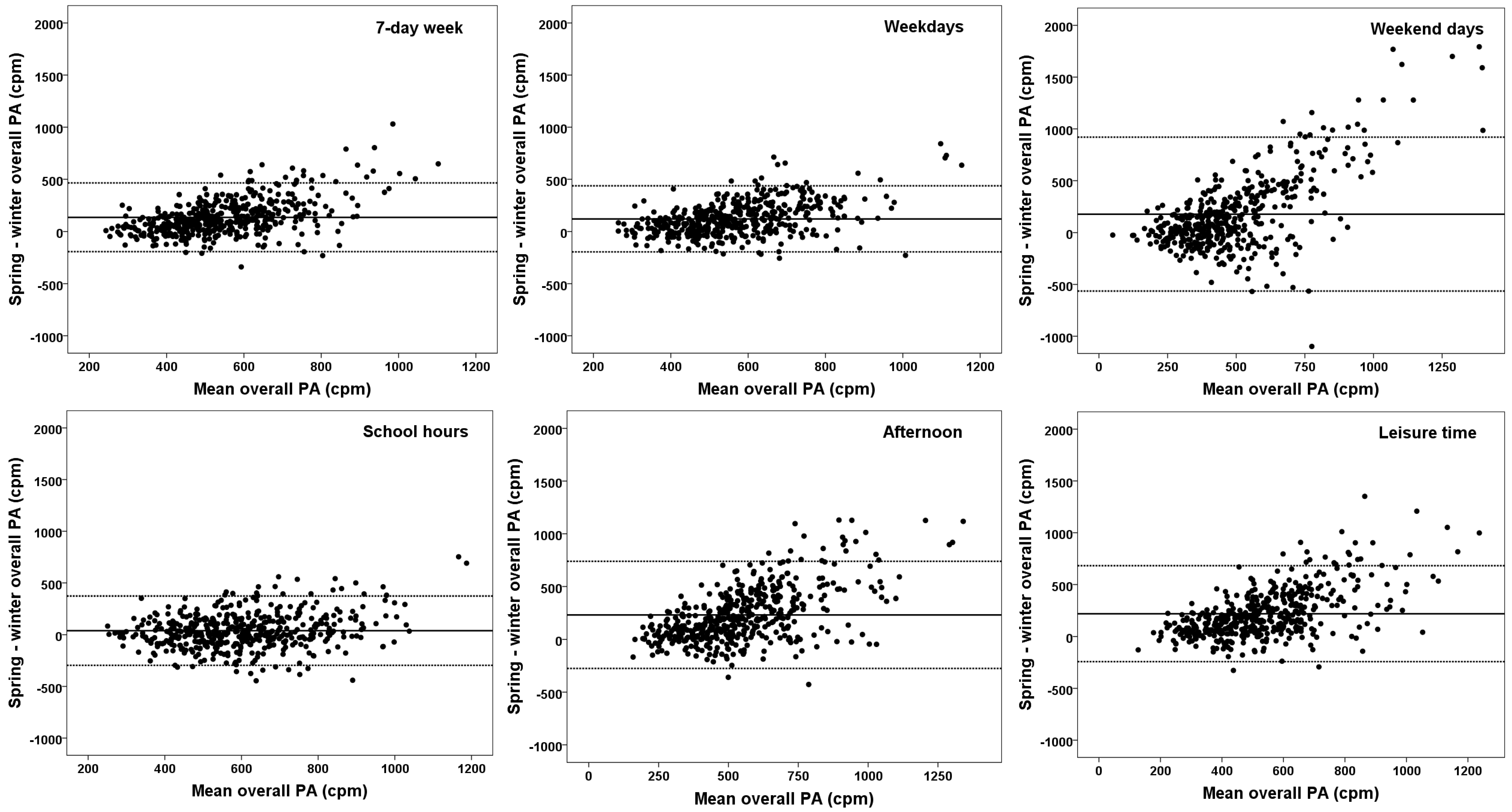

Supplement: Supplementary file 1 — Figure S1. Bland Altman plots of agreement for overall physical activity level (cpm) for different domains over two consecutive weeks of measurement. Bland Altman plots (mean of two weeks of measurement on the x-axis versus the difference between them on the y-axis) for a 7-day week, weekdays, weekend days, school hours, afternoon and total leisure time. All results are based on n = 465 children. The full line is the bias between weeks, whereas the dotted lines are 95% limits of agreement. (TIFF 4673 kb) [file 12889_2018_5743_MOESM1_ESM.tiff]

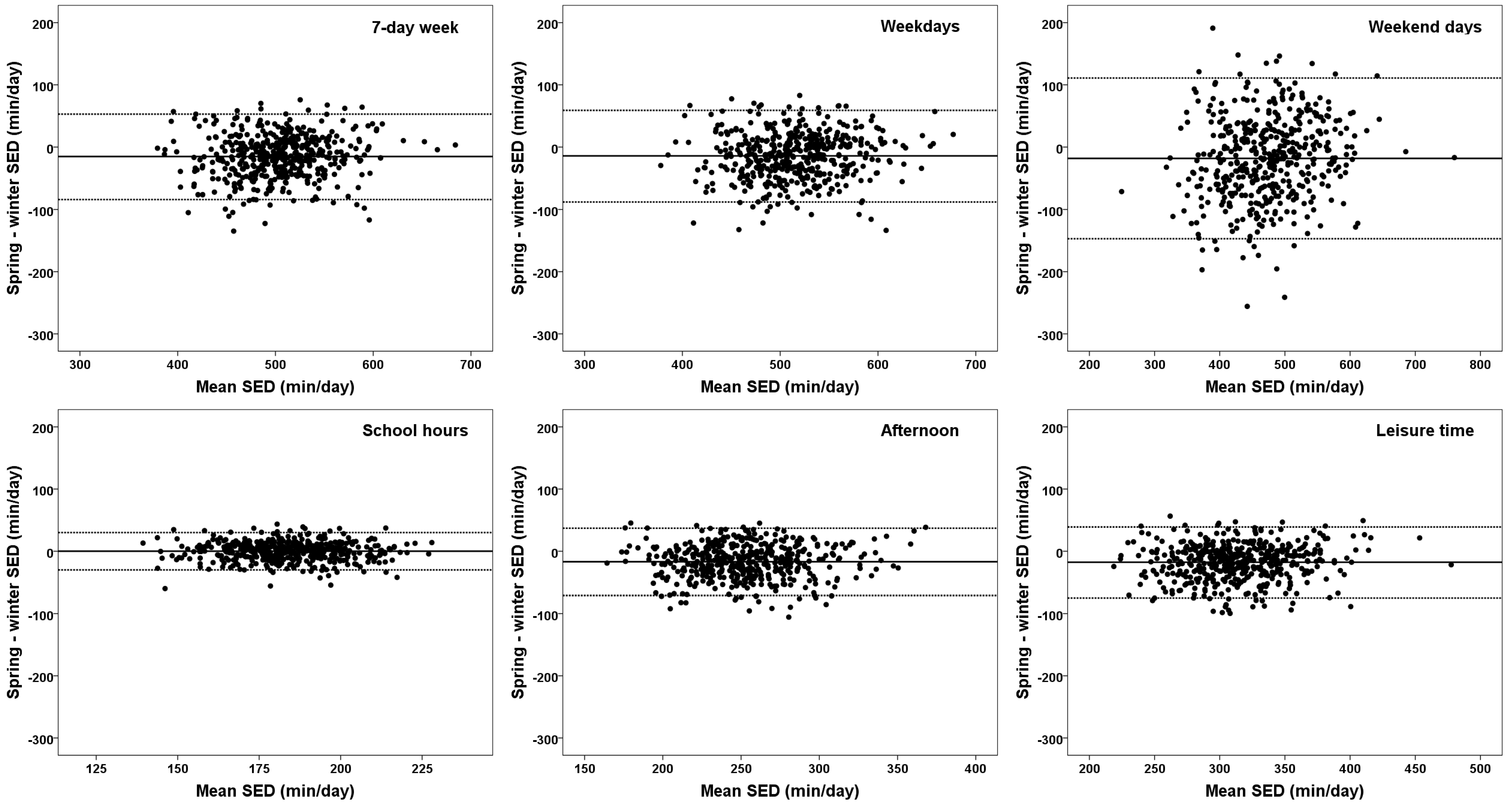

Supplement: Supplementary file 2 — Figure S2. Bland Altman plots of agreement for sedentary time (min/day) for different domains over two consecutive weeks of measurement. Bland Altman plots (mean of two weeks of measurement on the x-axis versus the difference between them on the y-axis) for a 7-day week, weekdays, weekend days, school hours, afternoon and total leisure time. All results are based on n = 465 children. The full line is the bias between weeks, whereas the dotted lines are 95% limits of agreement. Please be aware that variability is not directly comparable between full days and part of days, due to different wear time. (TIFF 4520 kb) [file 12889_2018_5743_MOESM2_ESM.tiff]

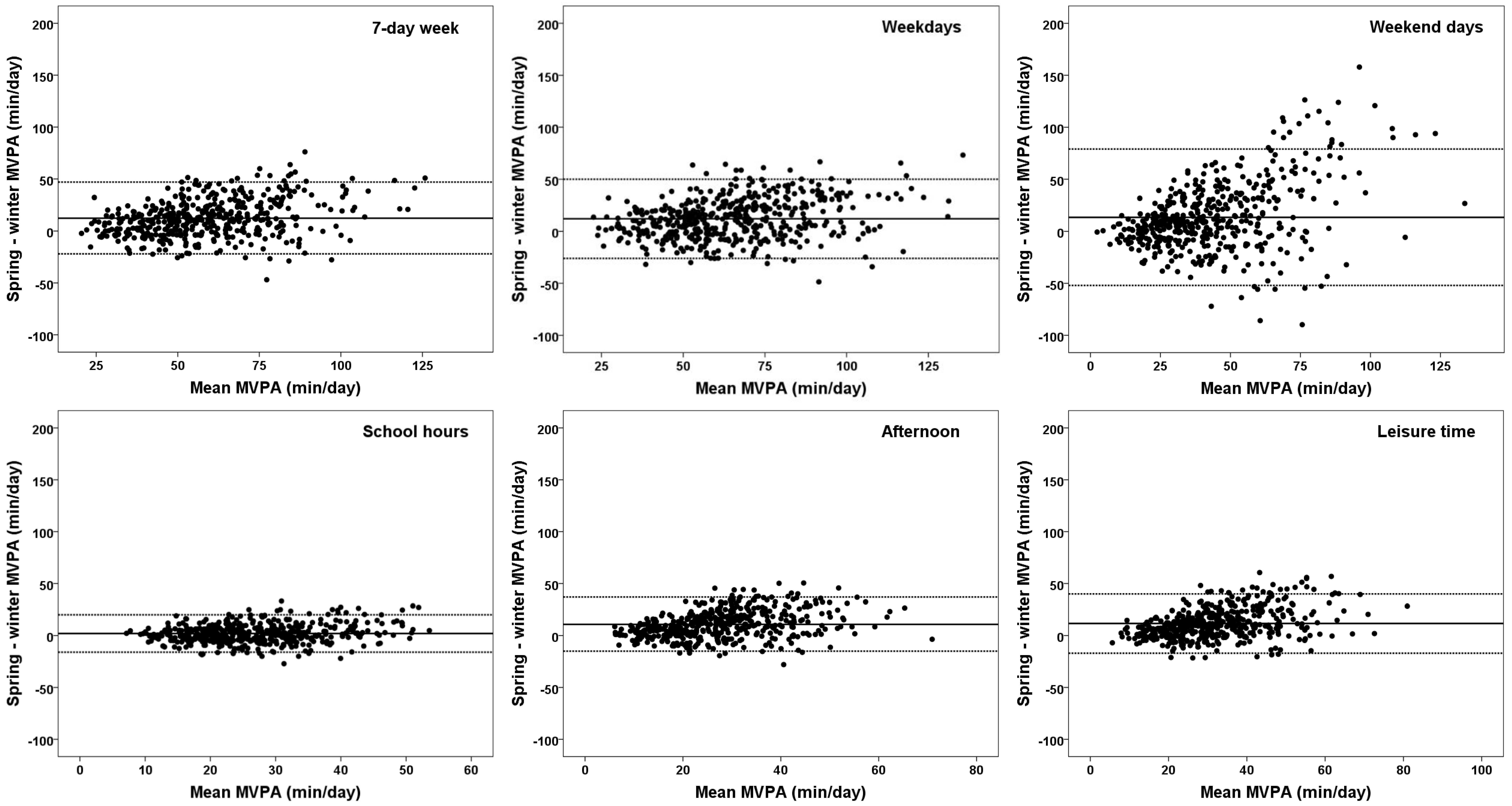

Supplement: Supplementary file 3 — Figure S3. Bland Altman plots of agreement for moderate-to-vigorous physical activity (min/day) for different domains over two consecutive weeks of measurement. Bland Altman plots (mean of two weeks of measurement on the x-axis versus the difference between them on the y-axis) for a 7-day week, weekdays, weekend days, school hours, afternoon and total leisure time. All results are based on n = 465 children. The full line is the bias between weeks, whereas the dotted lines are 95% limits of agreement. Please be aware that variability is not directly comparable between full days and part of days, due to different wear time. (TIFF 5467 kb) [file 12889_2018_5743_MOESM3_ESM.tiff]
