# Supplementary material for: Reproducibility of domain-specific physical activity over two seasons in children
Source: BMC Public Health. 2018 Jul 3;18:821. doi: 10.1186/s12889-018-5743-8 (PMC6029381; doi:10.1186/s12889-018-5743-8)
Supplement: Supplementary file 4 — Table S1. Differences (spring – winter) in physical activity level between seasons across all physical activity outcomes. (DOCX 16 kb) [file 12889_2018_5743_MOESM4_ESM.docx]

**Additional file**

Additional file 4: **Table S1***. Differences (spring – winter) in physical activity level between seasons across all physical activity outcomes.*

| **Physical activity level** | **7-d Week** | **Weekdays** | **Weekend** | **School** | **Afternoon** | **Leisure** |
| --- | --- | --- | --- | --- | --- | --- |
|  | **Mean difference (95% confidence interval)** | | | | | |
| **Overall PA (cpm)** | 136  (121–152) | 121  (107–136) | 178  (143–213) | 39  (23–54) | 232  (209–256) | 220  (199–242) |
| **SED (min/day)** | -15.2  (-18.4–-11.9) | -14.2  (-17.6–-10.8) | -18.2  (-24.3–-12.0) | 0.1  (-1.3–1.5) | -16.9  (-19.4–-14.3) | -17.7  (20.4–-14.9) |
| **LPA (min/day)** | 2.8  (0.5–5.1) | 2.1  (-0.3–4.6) | 4.7  (0.4–8.9) | -2.1  (-3.2–-1.1) | 6.0  (4.3–7.8) | 5.9  (4.0–7.8) |
| **MPA (min/day)** | 4.0  (3.1–4.9) | 3.9  (2.9–4.9) | 4.5  (2.8–6.2) | 0.4  (0.0–0.9) | 4.3  (3.7–5.0) | 4.5  (3.7–5.2) |
| **VPA (min/day)** | 8.3  (7.3–9.3) | 8.2  (7.2–9.2) | 8.9  (7.1–10.7) | 1.0  (0.4–1.5) | 6.4  (5.7–7.2) | 7.2  (6.4–8.0) |
| **MVPA (min/day)** | 12.4  (10.7–14.0) | 12.1  (10.4–13.9) | 13.5  (10.4–16.6) | 2.0  (1.2–2.9) | 10.8  (9.6–12.0) | 11.7  (10.4–13.1) |
|  | **Effect size (mean difference/pooled SD)** | | | | | |
| **Overall PA (cpm)** | 0.75 | 0.67 | 0.60 | 0.21 | 0.87 | 0.91 |
| **SED (min/day)** | -0.29 | -0.26 | -0.22 | 0.01 | -0.42 | -0.39 |
| **LPA (min/day)** | 0.08 | 0.06 | 0.10 | -0.16 | 0.27 | 0.23 |
| **MPA (min/day)** | 0.36 | 0.33 | 0.30 | 0.07 | 0.61 | 0.56 |
| **VPA (min/day)** | 0.64 | 0.59 | 0.56 | 0.14 | 0.80 | 0.80 |
| **MVPA (min/day)** | 0.56 | 0.50 | 0.48 | 0.20 | 0.77 | 0.73 |

PA = physical activity; cpm = counts per minute; SED = sedentary time; LPA = light physical activity; MPA = moderate physical activity; VPA = vigorous physical activity; MVPA = moderate-to-vigorous physical activity; all results are based on n = 465 children.
