# Supplementary material for: Reproducibility of domain-specific physical activity over two seasons in children
Source: BMC Public Health. 2018 Jul 3;18:821. doi: 10.1186/s12889-018-5743-8 (PMC6029381; doi:10.1186/s12889-018-5743-8)
Supplement: Supplementary file 5 — Table S2a. The reliability for different outcome variables for one out of two weeks of measurement for a 7-d week, weekdays and weekend days, applying a ≥ 4 weekdays and 2 weekend days wear time criterion (n = 257 (38%) children). Table S2b. The reliability for different outcome variables for one out of two weeks of measurement for school hours, afternoon and total leisure time, applying a ≥ 4 weekdays and 2 weekend days wear time criterion (n = 257 (38%) children). (DOCX 20 kb) [file 12889_2018_5743_MOESM5_ESM.docx]

Additional file

Additional file 5: **Table S2a**. *The reproducibility of different outcome variables for one out of two weeks of measurement for a 7-d week, weekdays and weekend days, applying a ≥ 4 weekdays and 2 weekend days wear time criterion (n = 257 (38%) children).*

|  | **Week** | | | **Weekdays** | | | **Weekend** | | |
| --- | --- | --- | --- | --- | --- | --- | --- | --- | --- |
|  | **ICC_s_** | **LoA** | **N** | **ICC_s_** | **LoA** | **N** | **ICC_s_** | **LoA** | **N** |
|  | **Not corrected for season (absolute agreement definition)** | | | | | | | | |
| **Overall PA (cpm)** | 0.29 | 420 | 9.9 | 0.40 | 380 | 6.0 | <0.01 | 810 | - |
| **SED (min/day)** | 0.65 | 67.5 | 2.1 | 0.62 | 73.9 | 2.5 | 0.41 | 117 | 5.8 |
| **LPA (min/day)** | 0.69 | 47.3 | 1.8 | 0.62 | 53.6 | 2.4 | 0.52 | 78.5 | 3.7 |
| **MPA (min/day)** | 0.60 | 18.9 | 2.6 | 0.58 | 21.2 | 2.8 | 0.32 | 30.6 | 8.6 |
| **VPA (min/day)** | 0.44 | 25.4 | 5.2 | 0.50 | 26.1 | 4.0 | 0.08 | 40.4 | 45.3 |
| **MVPA (min/day)** | 0.56 | 39.0 | 3.1 | 0.58 | 41.5 | 2.9 | 0.21 | 63.6 | 15.2 |
|  | **Corrected for season (consistency definition)** | | | | | | | | |
| **Overall PA (cpm)** | 0.51 | 322 | 3.9 | 0.57 | 302 | 3.0 | 0.12 | 717 | 30.1 |
| **SED (min/day)** | 0.69 | 62.7 | 1.8 | 0.65 | 70.3 | 2.2 | 0.44 | 113 | 5.1 |
| **LPA (min/day)** | 0.69 | 47.4 | 1.8 | 0.62 | 53.7 | 2.4 | 0.52 | 78.4 | 3.7 |
| **MPA (min/day)** | 0.65 | 17.5 | 2.2 | 0.62 | 20.0 | 2.4 | 0.34 | 29.7 | 7.7 |
| **VPA (min/day)** | 0.62 | 19.6 | 2.5 | 0.65 | 21.1 | 2.2 | 0.20 | 35.6 | 15.6 |
| **MVPA (min/day)** | 0.69 | 31.2 | 1.8 | 0.69 | 34.7 | 1.8 | 0.30 | 57.7 | 9.5 |

PA = physical activity; cpm = counts per minute; SED = sedentary time; LPA = light physical activity; MPA = moderate physical activity; VPA = vigorous physical activity; MVPA = moderate-to-vigorous physical activity; ICC_s_ = intra-class correlation for a single week of measurement adjusted for wear time; N = number of weeks needed to achieve a ICC = 0.80; LoA = 95% limits of agreement

***Table S2b***. *The reproducibility of different outcome variables for one out of two weeks of measurement for school hours, afternoon and total leisure time, applying a ≥ 4 weekdays and 2 weekend days wear time criterion (n = 257 (38%) children).*

|  | **School** | | | **Afternoon** | | | **Leisure** | | |
| --- | --- | --- | --- | --- | --- | --- | --- | --- | --- |
|  | **ICC_s_** | **LoA** | **N** | **ICC_s_** | **LoA** | **N** | **ICC_s_** | **LoA** | **N** |
|  | **Not corrected for season (absolute agreement definition)** | | | | | | | | |
| **Overall PA (cpm)** | 0.58 | 318 | 2.9 | 0.18 | 677 | 18.6 | 0.16 | 619 | 21.6 |
| **SED (min/day)** | 0.62 | 28.1 | 2.4 | 0.45 | 58.5 | 5.0 | 0.54 | 60.5 | 3.4 |
| **LPA (min/day)** | 0.56 | 22.6 | 3.1 | 0.53 | 36.9 | 3.5 | 0.65 | 37.7 | 2.1 |
| **MPA (min/day)** | 0.55 | 9.8 | 3.3 | 0.41 | 14.9 | 5.8 | 0.45 | 16.0 | 5.0 |
| **VPA (min/day)** | 0.59 | 11.7 | 2.8 | 0.28 | 19.5 | 10.1 | 0.24 | 21.9 | 12.7 |
| **MVPA (min/day)** | 0.60 | 17.7 | 2.6 | 0.35 | 31.6 | 7.5 | 0.36 | 34.5 | 7.3 |
|  | **Corrected for season (consistency definition)** | | | | | | | | |
| **Overall PA (cpm)** | 0.59 | 314 | 2.8 | 0.42 | 518 | 5.5 | 0.42 | 462 | 5.5 |
| **SED (min/day)** | 0.62 | 28.1 | 2.4 | 0.54 | 51.6 | 3.4 | 0.62 | 53.1 | 2.4 |
| **LPA (min/day)** | 0.57 | 22.2 | 3.0 | 0.55 | 36.2 | 3.3 | 0.66 | 37.0 | 2.0 |
| **MPA (min/day)** | 0.55 | 9.8 | 3.3 | 0.53 | 12.9 | 3.6 | 0.54 | 14.1 | 3.4 |
| **VPA (min/day)** | 0.59 | 11.6 | 2.8 | 0.49 | 15.4 | 4.2 | 0.46 | 16.9 | 4.7 |
| **MVPA (min/day)** | 0.61 | 17.4 | 2.5 | 0.55 | 24.6 | 3.3 | 0.55 | 27.0 | 3.3 |

PA = physical activity; cpm = counts per minute; SED = sedentary time; LPA = light physical activity; MPA = moderate physical activity; VPA = vigorous physical activity; MVPA = moderate-to-vigorous physical activity; ICC_s_ = intra-class correlation for a single week of measurement adjusted for wear time; N = number of weeks needed to achieve a ICC = 0.80; LoA = 95% limits of agreement; *Afternoon + Weekend
